# Mapping Knowledge Landscapes and Emerging Trends of Sarcopenic Obesity in Older Adults: A Bibliometric Analysis From 2004 to 2023

**DOI:** 10.7759/cureus.62300

**Published:** 2024-06-13

**Authors:** Ning Zhang, Xuan Qu, Haokang Zhou, Lin Kang

**Affiliations:** 1 Department of Geriatrics, Peking Union Medical College Hospital, Peking Union Medical College, Chinese Academy of Medical Sciences, Beijing, CHN; 2 Department of Internal Medicine, Peking Union Medical College Hospital, Peking Union Medical College, Chinese Academy of Medical Sciences, Beijing, CHN

**Keywords:** bibliometric analysis, citespace, vosviewer, older adult, obesity, sarcopenia

## Abstract

Background

The prevalence of obesity in combination with sarcopenia, the age-related loss of muscle mass and strength or physical function, is on the rise among adults aged 65 years and older. A significant portion of this demographic now falls under the classification of sarcopenic obesity, a high-risk geriatric syndrome predominantly seen in an aging population vulnerable to compounded complications from both sarcopenia and obesity. It is essential to promptly evaluate the impact of academic research in this field, taking into account factors such as geographical regions, authors, journals, and institutions. Furthermore, exploring current topics and identifying potential areas that could inspire future researchers to conduct additional studies is crucial for advancing overall health in this population.

Methodology

A search was conducted in the Web of Science Core Collection database to identify English language articles and reviews focusing on sarcopenic obesity in older adults, published between January 1, 2004, and December 31, 2023. Bibliometric analysis was performed using VOSviewer (v.1.6.18) and CiteSpace (v.6.1.R2).

Results

A total of 985 original English-language articles were collected, consisting of 783 articles and 202 reviews. The volume of research publications in this field has shown significant growth since 2012. The United States leads in contributions, with 239 articles (24.3% of the total) and the highest number of citations at 18,403, along with the highest total link strength. The University of Melbourne in Australia stands out with 25 published articles (2.5% of the total). University of Verona in Italy has the most citations at 9,405, and Monash University in Australia has the highest total link strength at 53. Among prolific authors, John A. Batsis from University of North Carolina at Chapel Hill is the most productive with 24 articles (2.4% of the total). The journal "Nutrients" has the most articles on sarcopenic obesity in older adults, publishing 54 articles (5.5% of the total). Key topics in this area include sarcopenia, obesity, sarcopenic obesity, and elderly. Recent interventions focus on "nutrition" and "exercise" for sarcopenic obesity in older adults.

Conclusions

Research on sarcopenic obesity in older adults has seen significant growth on a global scale from 2004 to 2023, indicating a promising area for further study with potential benefits from current advancements. Although academic inquiries have shed light on various aspects of sarcopenic obesity in older adults, there remains a noticeable dearth of clinical research and evidence-based medicine on the effective management of this condition in elderly individuals. Future studies could focus on developing tailored interventions for older adults with sarcopenic obesity.

## Introduction

The prevalence of sarcopenia and obesity has been on the rise due to global urbanization, aging, and changes in lifestyles, making it a significant public health concern worldwide. Sarcopenia, initially characterized by age-related decline in skeletal muscle mass (SMM) and strength, now encompasses factors like chronic diseases, physical inactivity, movement disorders, and poor nutrition that contribute to muscle loss. On the other hand, obesity is a chronic condition marked by a substantial increase in total adipose tissue and/or visceral fat mass, leading to detrimental health effects. Severe cases of sarcopenia and obesity together exacerbate metabolic disorders and elevate the risk of adverse outcomes such as falls, disability, and fractures. This combination is also linked to cardiovascular disease, diabetes, non-alcoholic fatty liver disease, and other metabolic conditions, influencing their onset, progression, and mortality [1]. The co-occurrence of sarcopenia and obesity, known as sarcopenic obesity (SO), was initially coined by Baumgartner in 2000 to characterize a state where both conditions coexist [2]. Research suggests that approximately 11% of older adults worldwide have SO, with prevalence notably rising after the age of 70 years [3].

Currently, there is a lack of standardized diagnostic criteria for SO. Furthermore, factors such as age and race can impact body composition, specifically muscle mass and body fat content, resulting in the heterogeneous diagnosis of SO. The prevailing diagnostic criteria primarily focus on the combination of sarcopenia and obesity. Sarcopenia diagnosis involves distinguishing between primary and secondary sarcopenia based on the underlying cause. Primary sarcopenia is primarily associated with age-related changes and declining bodily functions, while secondary sarcopenia can be linked to various conditions such as inflammatory diseases, osteoarthritis, neurological disorders, reduced physical activity, malnutrition, and overnutrition/obesity. The assessment of sarcopenia typically considers three key variables: SMM, muscle strength, and physical performance. Variations in diagnostic criteria for sarcopenia exist due to differences in regions and ethnic groups. The 2018 European Working Group on Sarcopenia in the Elderly recommended that the diagnosis of sarcopenia in the elderly be based on walking speed, grip strength, and appendicular skeletal muscle index (ASMI). ASMI is calculated as limb SMM (kg) divided by height (m) squared. Sarcopenia is diagnosed if the ASMI value is more than 2 standard deviations below the mean reference value for the young population [4]. The Asian Working Group for Sarcopenia (AWGS) 2014 consensus defined sarcopenia as the age-related loss of muscle mass, along with low muscle strength and/or low physical performance. The group specified cutoffs for each diagnostic component. In 2019, AWGS maintained the same definition of sarcopenia but made revisions to the diagnostic algorithm, protocols, and certain criteria. Low muscle strength is now defined as handgrip strength below 28 kg for men and 18 kg for women. Criteria for low physical performance include a six-meter walk speed below 1.0 m/s, a Short Physical Performance Battery score of 9 or lower, or a five-time chair stand test taking 12 seconds or longer. The original cutoffs for height-adjusted muscle mass remain unchanged: less than 7.0 kg/m^2^ for men using dual-energy X-ray absorptiometry (DXA) and less than 5.4 kg/m^2^ for women, as well as less than 7.0 kg/m^2^ for men and less than 5.7 kg/m^2^ for women using bioimpedance. Furthermore, the AWGS 2019 update introduces distinct algorithms for community and hospital settings, both of which start with screening calf circumference (below 34 cm in men and below 33 cm in women), SARC-F (strength, assistance in walking, rising from a chair, climbing stairs, and falls) score of 4 or higher, or SARC-CalF (strength, assistance in walking, rising from a chair, climbing stairs, falls, and calf circumference) score of 11 or higher to enable early identification of individuals at risk for sarcopenia [5]. It is widely acknowledged by scholars that the reduction of SMM and muscle mass plays a crucial role in the clinical diagnosis of sarcopenia, with muscle strength being considered the most reliable indicator of muscle function. The diagnosis of obesity involves a variety of methods such as BMI, waist circumference, DXA, ultrasound, CT, or bioelectrical impedance analysis (BIA) for assessing body fat percentage, with BMI being the most commonly utilized indicator.

SO can develop subtly, with sarcopenia being easily concealed by obesity, underscoring the importance of early detection. To identify sarcopenia in patients at an early stage, it is essential to rely on a combination of multiple indicators rather than a single diagnostic criterion for improved accuracy. Screening and diagnosis of individuals suspected of having SO involve assessing an increase in BMI or waist circumference that exceeds race-specific obesity diagnostic thresholds, along with surrogate markers of sarcopenia (such as clinical symptoms or risk factors) or the completion of questionnaires (e.g., the simple five-item rating questionnaire for elderly patients) [6]. In current literature, obesity in the context of SO is commonly defined as a BMI of ≥ 30 kg/m^2^ and an elevated body fat percentage (≥27% or 28% for men and ≥28% for women), or specific thresholds (e.g., 35%, 38%, or 40%). Additionally, waist circumference exceeding population-specific tertiles or the World Health Organization's defined cut-off values (≥88 cm for women and ≥102 cm for men) are frequently utilized [7].

Existing studies have shown that the presence of SO is linked to a range of negative clinical outcomes. These include metabolic issues such as dyslipidemia, diabetes mellitus, metabolic syndrome, insulin resistance, and reduced levels of vitamin D. Additionally, this condition is associated with geriatric syndromes like cognitive impairment, functional limitations, increased risk of falls, depressive symptoms, dementia, frailty, osteoporosis, short sleep duration, low physical activity levels, fatigue, and disability. Furthermore, SO can impact cancer outcomes and treatment by reducing overall, recurrence-free, and disease-free survival rates, increasing surgical complications and hospital length of stay, and decreasing tolerance to therapy. It also heightens the risk of mortality due to various causes, such as cardiovascular disease, heart failure, and cardiovascular surgery, as well as morbidity outcomes like hypertension, lung diseases, stroke, and arthritis. Moreover, SO is associated with the development of other clinical conditions, such as hospitalization, poor nutritional status, limited improvement in activities of daily living, dysphagia after stroke, reduced quality of life, inflammation, and poor recovery in knee flexion range of motion after total knee replacement [8]. Despite its significant importance, clinicians still lack a comprehensive understanding of SO. This article conducts a bibliometric analysis of studies related to SO in older adults from 2004 to 2023. The objective is to identify development trends, research status, and hotspots in this field, ultimately enhancing clinicians' awareness of current research on SO in older adults. By providing insights from the existing literature, this analysis aims to improve clinicians' comprehension and management skills in dealing with SO in the elderly.

## Materials and methods

Ethics

The study was reviewed and approved by the Ethics Committee of Peking Union Medical College Hospital (approval number: I-23PJ738).

Data sources and search strategies

The study utilized data exclusively from the Web of Science Core Collection database, which includes academic publications from 250 global fields [9]. This database is commonly used by researchers for bibliometric analysis. The data encompass literature published between January 1, 2004, and December 31, 2023. The search query used was: TS=('the aged' OR 'the elderly' OR 'older adult' OR 'older patient' OR 'geriatric' OR 'older') AND T=('Sarcopenic Obesity'), excluding letters, reviews, conference abstracts, and other document types. Only articles or review articles in English were included in the analysis, resulting in a total of 985 relevant documents, comprising 783 articles and 202 reviews. These documents were sourced from 79 countries/regions, 324 journals, and 4,006 institutions, and were authored by 5,168 unique authors.

Data collection and bibliometric analysis

This study utilized various tools for bibliometric analysis, such as Microsoft Office Excel 2021 (Microsoft Corporation, Redmond, WA), VOSviewer (v.1.6.18), CiteSpace (v.6.1.R6), and the R package "bibliometrix" for data analysis and visualization [10]. The relevant information, including titles, authors, keywords, affiliations, countries/regions, citations, journal details, and publication dates, was extracted from the Web of Science Core Collection database for a total of 2590 documents. Subsequently, the processed data were imported into VOSviewer, CiteSpace, and the R package "bibliometrix" for bibliometric analysis. CiteSpace was utilized to create knowledge maps specific to the field and employ dynamic network analysis techniques to examine hot topics, trends, and frontiers within the scientific domain. Specifically, CiteSpace was used for co-occurrence and clustering analyses related to authorship, research institutions, and countries. On the other hand, VOSviewer extracted bibliographic networks (co-authorship, co-occurrence, and citation-based) from the data to analyze collaborative relationships among countries, authors, and institutions, as well as co-occurrence relationships among keywords. Bibliometrix, an open-source R package developed by Massimo Aria and Corrado Cuccurullo, was employed for comprehensive bibliometric analyses, particularly focusing on analyzing the evolving trends in keywords within the literature.

## Results

Publication and citation analysis

**Figure 1:**
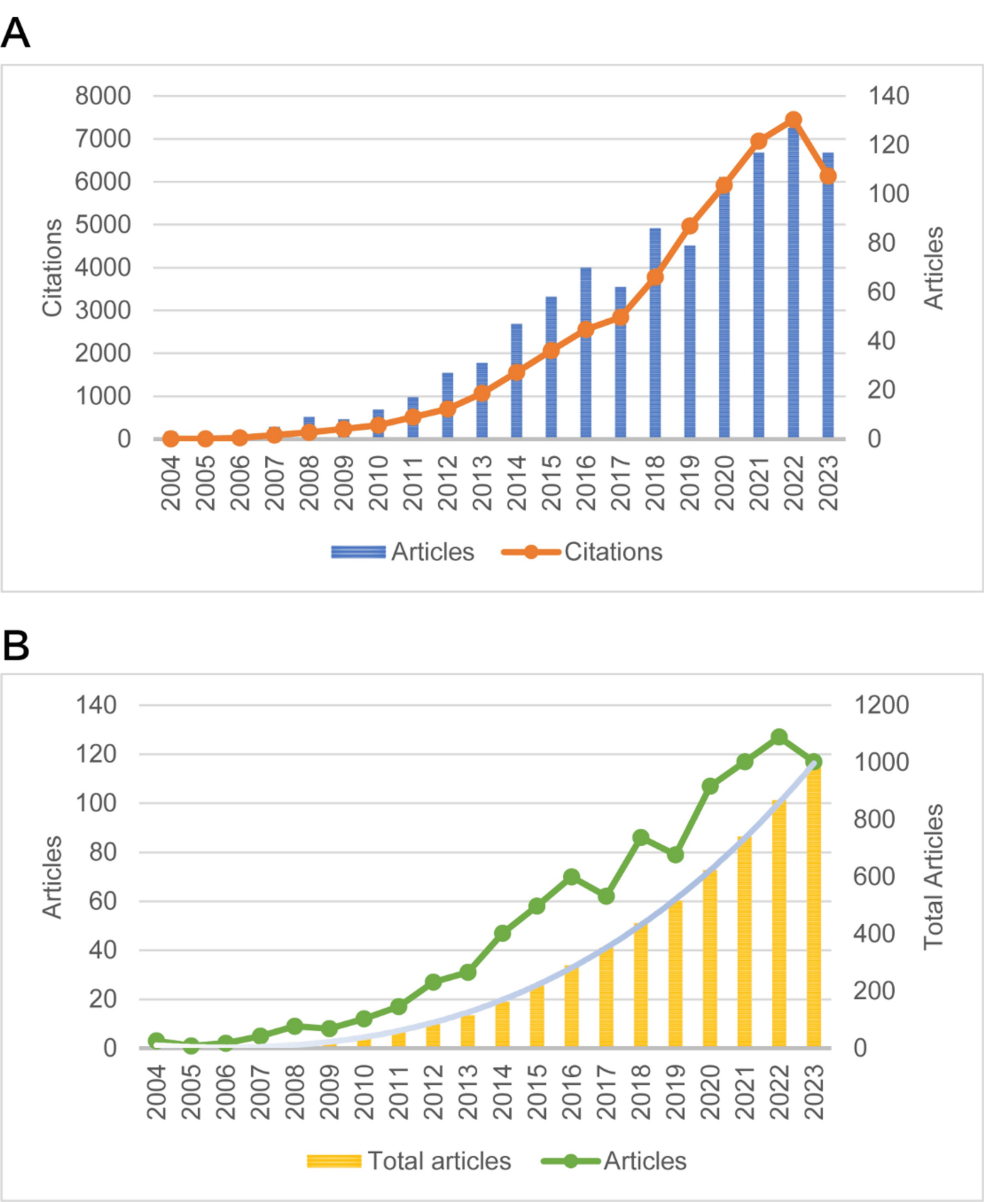
The analysis of annual publication quantity and citation frequency in the field of sarcopenic obesity in the elderly. (A) The annual publication quantity and citation frequency of research on sarcopenic obesity in the elderly from 2004 to 2023. (B) The annual publication quantity, cumulative publication quantity, and their polynomial fitting curves for sarcopenic obesity in the elderly from 2004 to 2023.

The research field of SO in the elderly has seen an increase in both publication volume and citation count over the past 20 years, as depicted in Figure 1. By the end of 2023, there was a notable rise in publication volume in 2016 and 2018, with a peak of 127 articles in 2022. Similarly, the citation frequency experienced rapid growth between 2017 and 2021, reaching a peak of 7,450 in 2022. Additionally, a cumulative publication volume analysis was conducted, and the polynomial fitting curve shown by the blue line in Figure 1B resulted in the fitting equation, y = 0.0927x^3 ^+ 1.0327x^2 ^- 8.7716x + 17.794, with a high fitting goodness (R²) of 0.9996.

Countries/regions analysis

Countries/regions play a vital role in determining the origins of publications and offer valuable insights into the regional and central aspects of SO research. In Table 1, rankings are provided based on the total publication volume, total citation count, and total link strength of the source countries/regions. The data highlight the USA as a leader in all three categories, showcasing its dominant position and significant contributions to SO research in the elderly. Following the USA, South Korea, China, and Italy have the next highest publication volumes. Italy stands out as a key international research focus, ranking second only to the USA in total citation count and total link strength.

**Table 1:** Ranking of the top 10 major countries/regions of sarcopenic obesity in elderly research from 2004 to 2023.

Rank	Countries/regions	Documents	Countries/regions	Citations	Countries/regions	Total link strength
1	USA	239	USA	18403	USA	230
2	South Korea	130	Italy	14002	Italy	196
3	China	89	England	13506	France	186
4	Italy	86	Germany	12184	Germany	157
5	England	80	France	12127	Spain	152
6	Japan	80	Sweden	10551	England	143
7	Brazil	71	Switzerland	10487	Sweden	136
8	Australia	70	Spain	10413	Netherlands	128
9	Spain	65	Belgium	9219	Brazil	124
10	Germany	63	South Korea	4965	Switzerland	121

**Figure 2:**
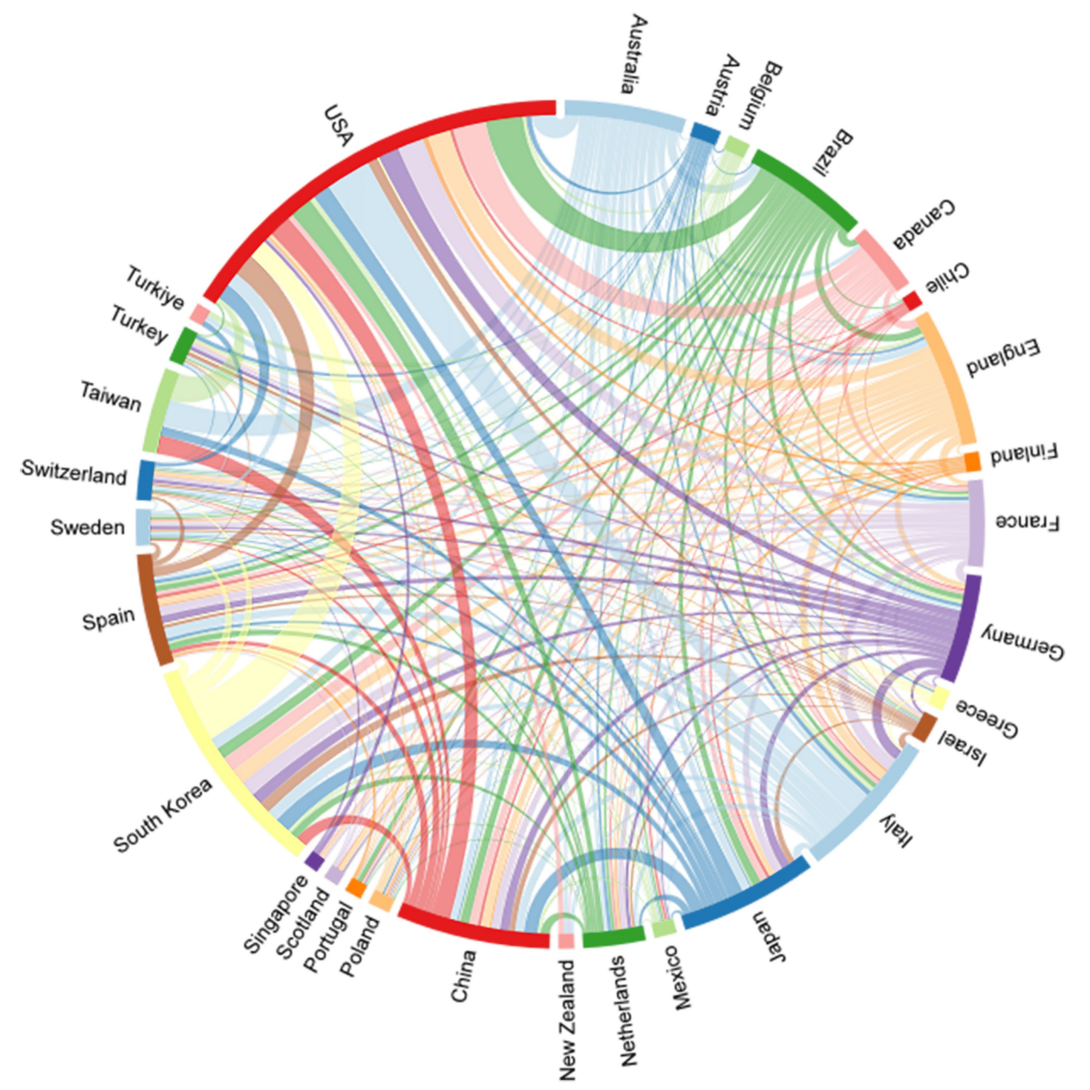
National/regional collaborative network mapping of sarcopenic obesity in elderly research from 2004 to 2023.

Figure 2, a chord diagram, visually represents the level of collaboration between countries/regions in a tangible manner. The colored lines connecting countries/regions depict collaborative relationships, with the widest red band indicating the most extensive collaborations, notably involving the USA. Italy is depicted as having close collaborations with the USA, as well as with other countries such as the Netherlands, China, and Germany. The diagram highlights that the primary collaborative endeavors are concentrated among countries/regions that play a significant role in SO research.

Author analysis

**Figure 3:**
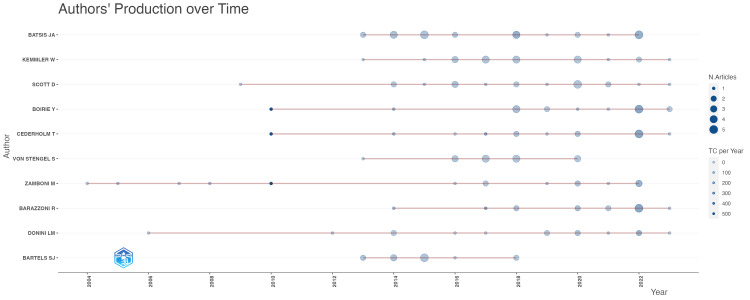
The annual publication trends of the top 10 authors in terms of publication volume are illustrated in the graph. The horizontal axis shows the progression of time, while the vertical axis displays the top 10 authors based on their publication volume. Node size indicates the quantity of published documents, and color depth represents the total citations (TC) per year, with darker colors indicating higher citation rates.

Author information is a crucial aspect of publication analysis, and Table 2 presents the top 10 authors based on publication volume, total citation count, total link strength, and co-citation count. Author co-citation analysis (ACA), as introduced by White and Griffith in 1981, serves as a key metric for this evaluation [11]. ACA measures how often one author is co-cited with another by third-party authors in a given publication, offering insights into research connections and directionality. The authors highlighted in Table 2 have made significant contributions to the field, with notable figures like Batsis, John A. (24 publications) and Kemmler, Wolfgang (21 publications). Batsis, John A. ranks highly in co-citation count and total link strength, indicating a strong influence. Figure 3 further illustrates the temporal distribution of publications for these authors, showing that Batsis, John A. has maintained a consistent level of productivity from 2014 to 2022.

**Table 2:** Ranking of the top 10 major authors of sarcopenic obesity in elderly research from 2004 to 2023.

Rank	Author	Documents	Total link strength	Countries/regions	Institution	Author	Co-citations	Total link strength	Countries/regions	Institution
1	Batsis, John A.	24	42	USA	University of North Carolina at Chapel Hill	Cruz-Jentoft, Aj	737	9387	Spain	Ramón y Cajal University Hospital
2	Kemmler, Wolfgang	21	57	Germany	Friedrich-Alexander-Universität Erlangen-Nürnberg	Baumgartner, RN	662	10378	Kentucky	University of Louisville
3	Scott, David	20	70	Australia	Deakin University	Batsis, JA	557	8413	USA	Duke University
4	Sieber, Cornel C.	17	48	Switzerland	University Hospital Basel	Janssen, I	477	7049	Canada	Queen's University
5	Von Stengel, Simon	15	43	Germany	Friedrich-Alexander-Universität Erlangen-Nürnberg	Stenholm, S	321	5314	Finland	University of Turku
6	Boirie, Yves	14	55	France	Clermont-Ferrand University Hospital	Kemmler, W	311	2851	Germany	Friedrich-Alexander-Universität Erlangen-Nürnberg
7	Bartels, Stephen J.	13	28	Turkey	Geisel School of Medicine at Dartmouth	Zamboni, M	305	4747	Italy	University of Verona
8	Cederholm, Tommy	12	39	Sweden	Uppsala University	Villareal, DT	290	5212	USA	Baylor College
9	Cruz-Jentoft, Alfonso	11	52	Spain	Ramón y Cajal University Hospital	Kim, TN	282	4347	Korea	Korea University
10	Donini, Lorenzo Maria	11	11	Italy	Sapienza University of Rome	Visser, M	279	4707	France	Université libre de Bruxelles

**Figure 4:**
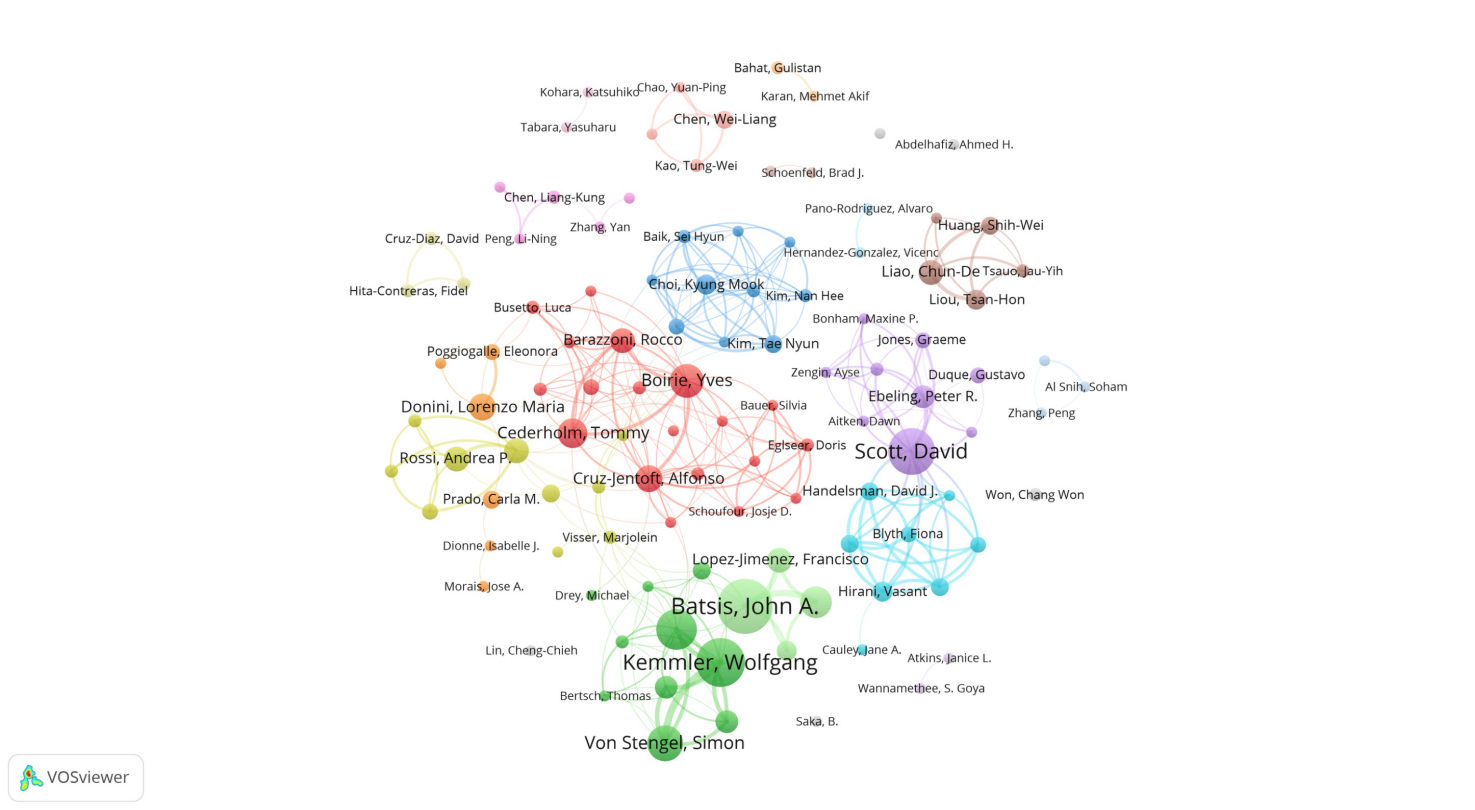
The map visualizes the co-occurring authors in research on sarcopenic obesity in the elderly. Nodes of different colors reflect authors in distinct clusters, with node size indicating the frequency of co-occurrence. The links depict the relationships among authors in terms of co-occurrence.

**Figure 5:**
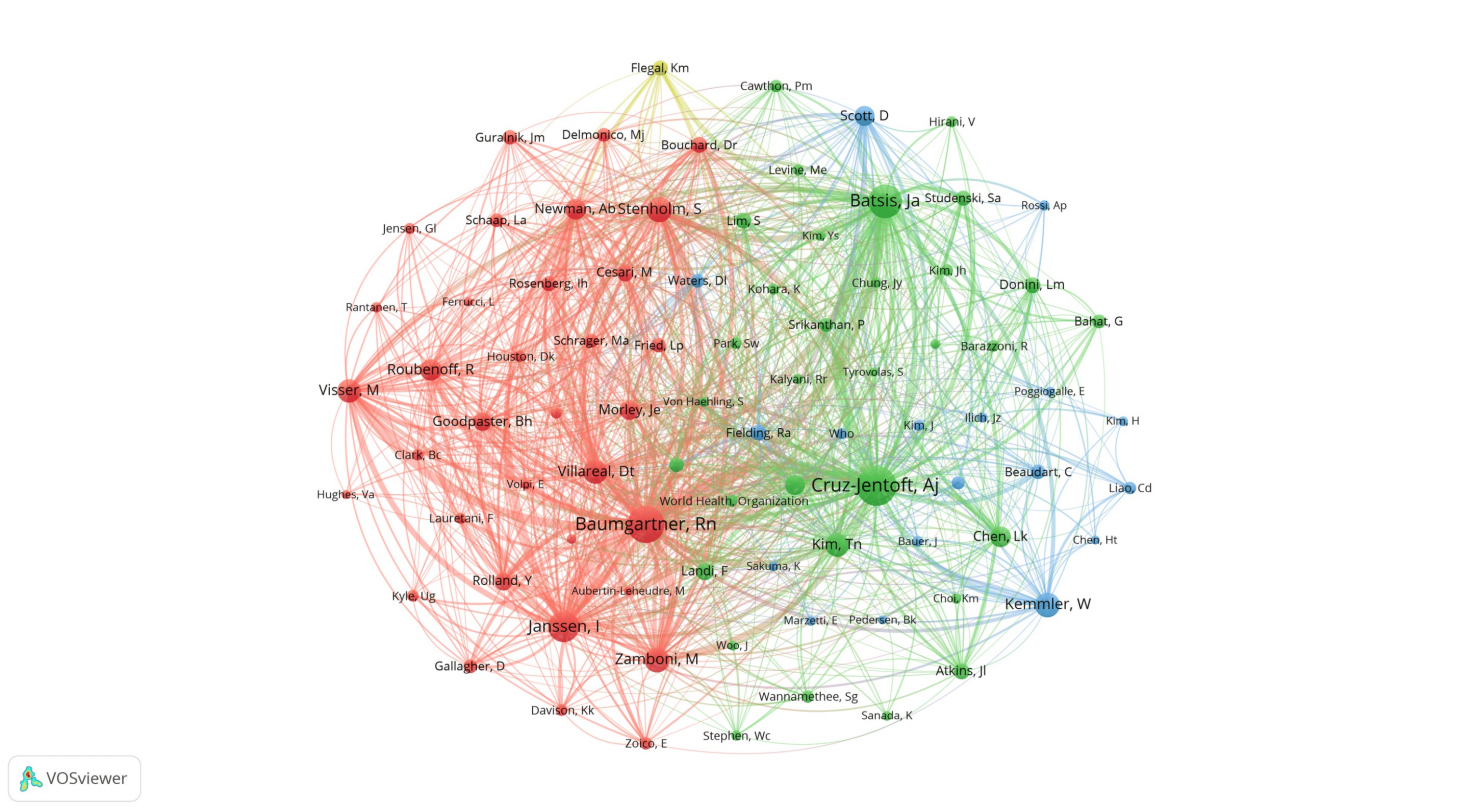
The map illustrates co-cited authors in research on sarcopenic obesity in the elderly, with node size indicating citation frequency. These data are visualized through VOSviewer to effectively depict and analyze the interconnections among cited authors in the research landscape of sarcopenic obesity in the elderly.

Figures 4, 5 illustrate the author collaboration network generated by VOSviewer. In Figure 4, the strength of collaboration relationships among authors is depicted, with co-citation relationships represented by lines connecting clusters. Authors within the same colored cluster signify higher research relevance. Notably, a strong transnational collaboration is observed between Batsis, John A. and Kemmler, Wolfgang. Additionally, significant collaboration exists between Scott, David and Ebeling, Peter R. Cross-national collaboration is also notable among authors from various European countries, such as Boirie, Yves, Cederholm, Tommy, Cruz-Jentoft, Alfonso, and Barazzoni, Rocco. Figure 5 highlights co-citation relationships, automatically grouping key authors into four major clusters based on research similarity and identifying key contributors within each cluster: Baumgartner, Rn (red cluster), Cruz-Jentoft, Alfonso, and Batsis, John A. (green cluster), Scott, David, and Kemmler, Wolfgang (blue cluster), and Flegal Km. and others.

Institution analysis

Table 3 displays the top 10 institutions ranked by publication volume, citation count, and total link strength. Leading in the number of publications are the University of Melbourne (25 articles) and Monash University (24 articles) from Australia. Seoul National University (21 articles) and Dartmouth-Hitchcock Medical Center (20 articles) closely follow with over 20 articles published in the field over the past two decades. Noteworthy for high citation counts are the University of Verona and Uppsala University, with the former also sharing the seventh position in publication volume with Dartmouth College.

**Table 3:** Ranking of the top 10 major institutions of sarcopenic obesity in elderly research from 2004 to 2023.

Rank	Institution	Publications	Original country	Institution	Total link strength	Original country	Institution	Citations	Original country
1	University of Melbourne	25	Australia	Monash University	53	Australia	University of Verona	9405	Italy
2	Monash University	24	Australia	Dartmouth–Hitchcock Medical Center	49	USA	Uppsala University	9063	Sweden
3	Seoul National University	21	Korea	Dartmouth College	46	USA	University of Erlangen–Nuremberg	8304	Germany
4	Dartmouth–Hitchcock Medical Center	20	USA	University of Melbourne	43	Australia	National Imaging Associates (NIA)	4637	USA
5	Korea University	19	Korea	Clermont Auvergne University	42	France	University of Pittsburgh	2008	USA
6	Yonsei University	18	Korea	Mayo Clinic	41	USA	Mayo Clinic	2005	USA
7	Dartmouth College	17	USA	University of Hohenheim	40	Germany	Seoul National University	1798	Korea
8	University of Verona	17	Italy	University of Trieste	39	Italy	Dartmouth–Hitchcock Medical Center	1793	USA
9	Istanbul University	16	Turkey	Geisel School of Medicine at Dartmouth	38	USA	Dartmouth College	1674	USA
10	Mayo Clinic	16	USA	University of Padua	37	Italy	Vrije Universiteit Amsterdam	1516	Netherlands

**Figure 6:**
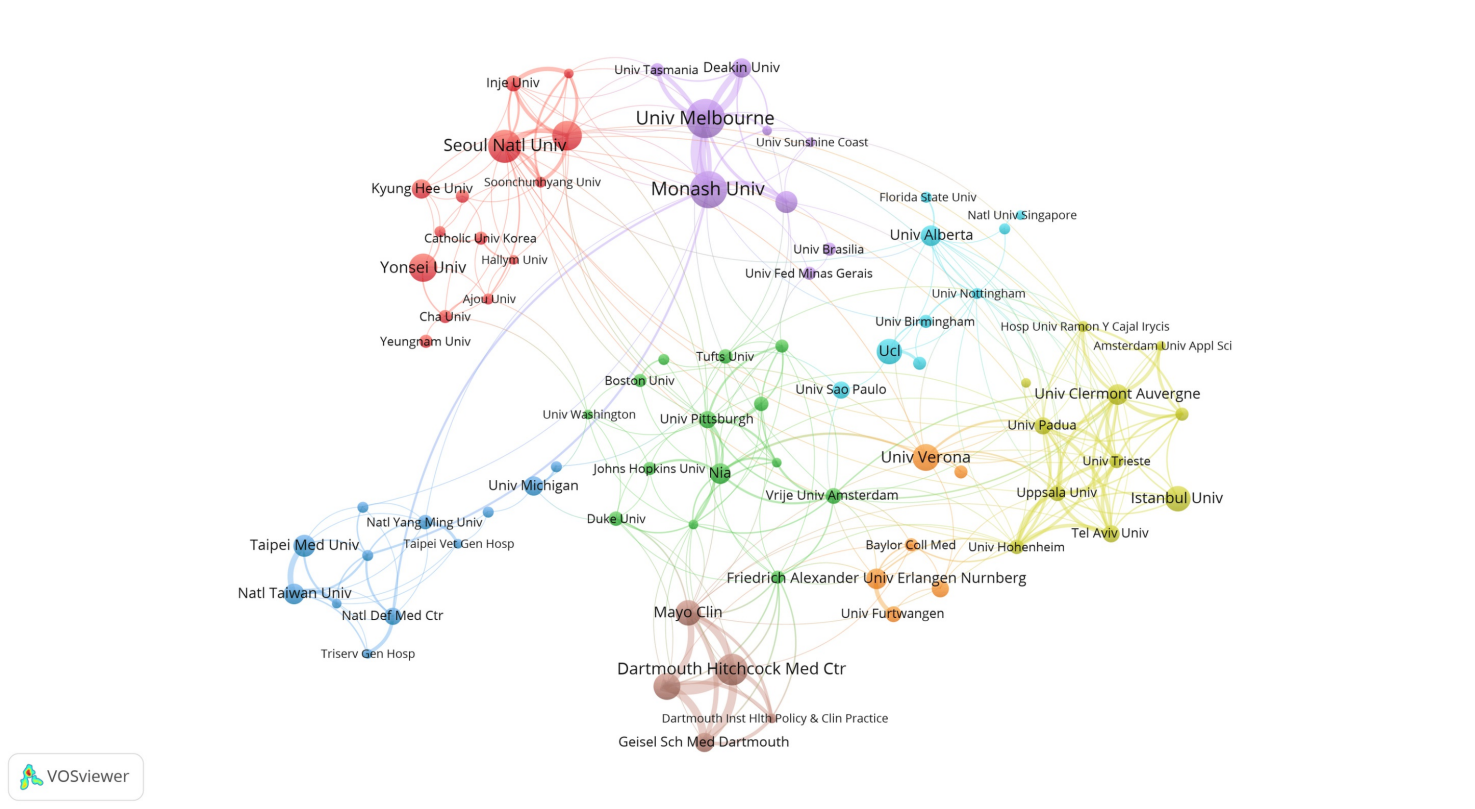
The analysis of institutions in the field of sarcopenic obesity in the elderly. The co-occurrence graph of research institutions is presented, with node size indicating the frequency of co-occurrence and connections representing the relationships between them. The size of each node reflects how frequently research institutions appear together, while the links indicate instances of their collaborative occurrences.

**Figure 7:**
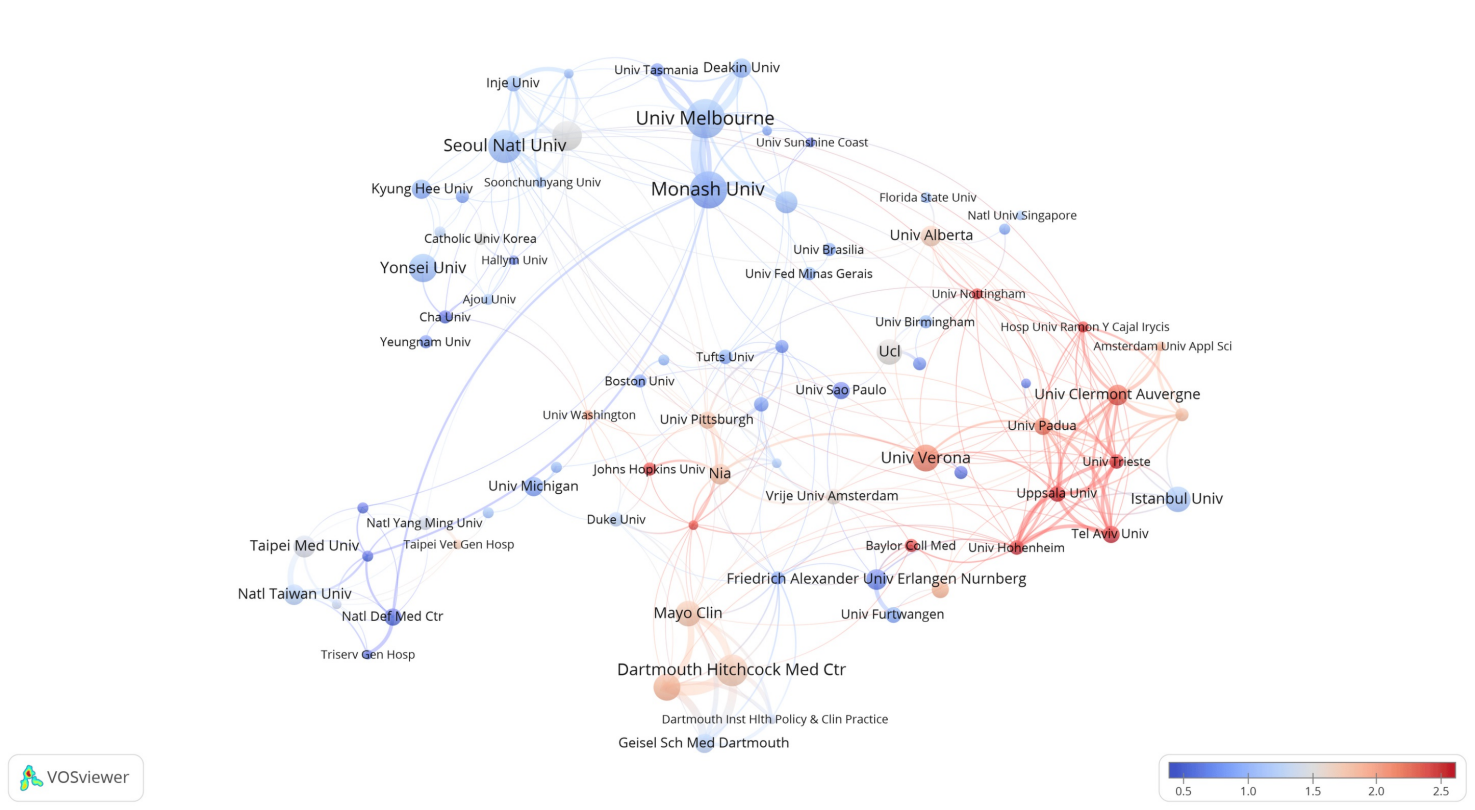
The analysis of institutions in the field of sarcopenic obesity in the elderly. The figure illustrates the recent contributions of institutions to research on sarcopenic obesity in the elderly compared to their overall output from 2004 to 2023. A red bias indicates increased influence, while a blue bias suggests decreased activity in the field. The color scale represents the ratio of keywords over the past five years, emphasizing institutions with significant impacts or reduced involvement in this study.

Figures 6, 7 demonstrate the strength of collaboration among institutions. In Figure 6, the clustering distribution reveals distinct regional patterns. Particularly, a close collaboration is evident between Australian institutions, i.e., the University of Melbourne and Monash University (purple cluster). This is followed by significant collaboration among institutions in the United States, including Dartmouth-Hitchcock Medical Center, Mayo Clinic, and Geisel School of Medicine at Dartmouth (brown cluster). Institutions in South Korea, centered around Seoul National University and Yonsei University, form a tightly interconnected network, involving many other Korean institutions (red cluster). Moreover, a unique cluster (yellow cluster) displays extensive collaboration among institutions from various countries, such as Uppsala University and Istanbul University. Moving on to Figure 7, it focuses on illustrating the chronological sequence of these collaboration relationships. As depicted, regional collaboration among countries/regions like South Korea, Australia, and Taiwan took place earlier, while emerging connections mainly involve institutions from different countries, such as Uppsala University and the University of Hohenheim.

Journal analysis

Tracing the sources of publications in the field of SO in the elderly to their respective journals is crucial for bibliometric analysis. This process helps readers identify relevant publication platforms and assists authors in making informed decisions when submitting manuscripts. It also sheds light on potential application domains and disciplines. Common evaluation indicators for journals include citation frequency, impact factor, and Journal Citation Reports (JCR) quartile classification. Table 4 presents the top 10 journals in terms of publication and citation counts in the field of SO in the elderly. Nutrients (54 articles) and Clinical Nutrition (33 articles) are the journals with the highest publication volume, both recognized as high-impact journals in JCR's Q1 category. Journals of Gerontology Series A: Biological Sciences and Medical Sciences (2,210 citations) and Journal of the American Geriatrics Society (2,070 citations) have each accumulated over 2,000 citations. Importantly, all the listed journals are classified in Q2 or higher, indicating that articles published in these journals hold significant scientific value and that the field itself garners considerable attention in society and the scientific community.

**Table 4:** Ranking of the top 10 major journals of sarcopenic obesity in elderly research from 2004 to 2023. JCR: Journal Citation Reports.

Journal	Publications	Impact factor (Journal Citation Reports 2022)	JCR quartile	Co-cited journal	Citations	Impact factor (Journal Citation Reports 2022)	JCR quartile
Nutrients	54	5.9	Q1	Journals of Gerontology Series A: Biological Sciences and Medical Sciences	2210	5.1	Q2
Clinical Nutrition	33	6.3	Q1	Journal of the American Geriatrics Society	2070	6.3	Q1
Experimental Gerontology	27	3.9	Q2	American Journal of Clinical Nutrition	1710	7.1	Q1
Journal of Nutrition Health and Aging	26	5.8	Q2	Clinical Nutrition	1192	6.3	Q1
BMC Geriatrics	23	4.1	Q2	Journal of the American Medical Directors Association	1102	7.6	Q1
Journal of Cachexia Sarcopenia and Muscle	22	8.9	Q1	Age and Ageing	1098	6.7	Q1
Archives of Gerontology and Geriatrics	21	4	Q2	International Journal of Obesity	945	4.9	Q2
Aging Clinical and Experimental Research	20	4	Q2	Journal of Nutrition Health and Aging	915	5.8	Q2
Journal of the American Medical Directors Association	20	7.6	Q1	Journal of Applied Physiology	896	3.3	Q2
PLoS One	20	3.7	Q2	PLoS One	842	3.7	Q2

**Figure 8:**
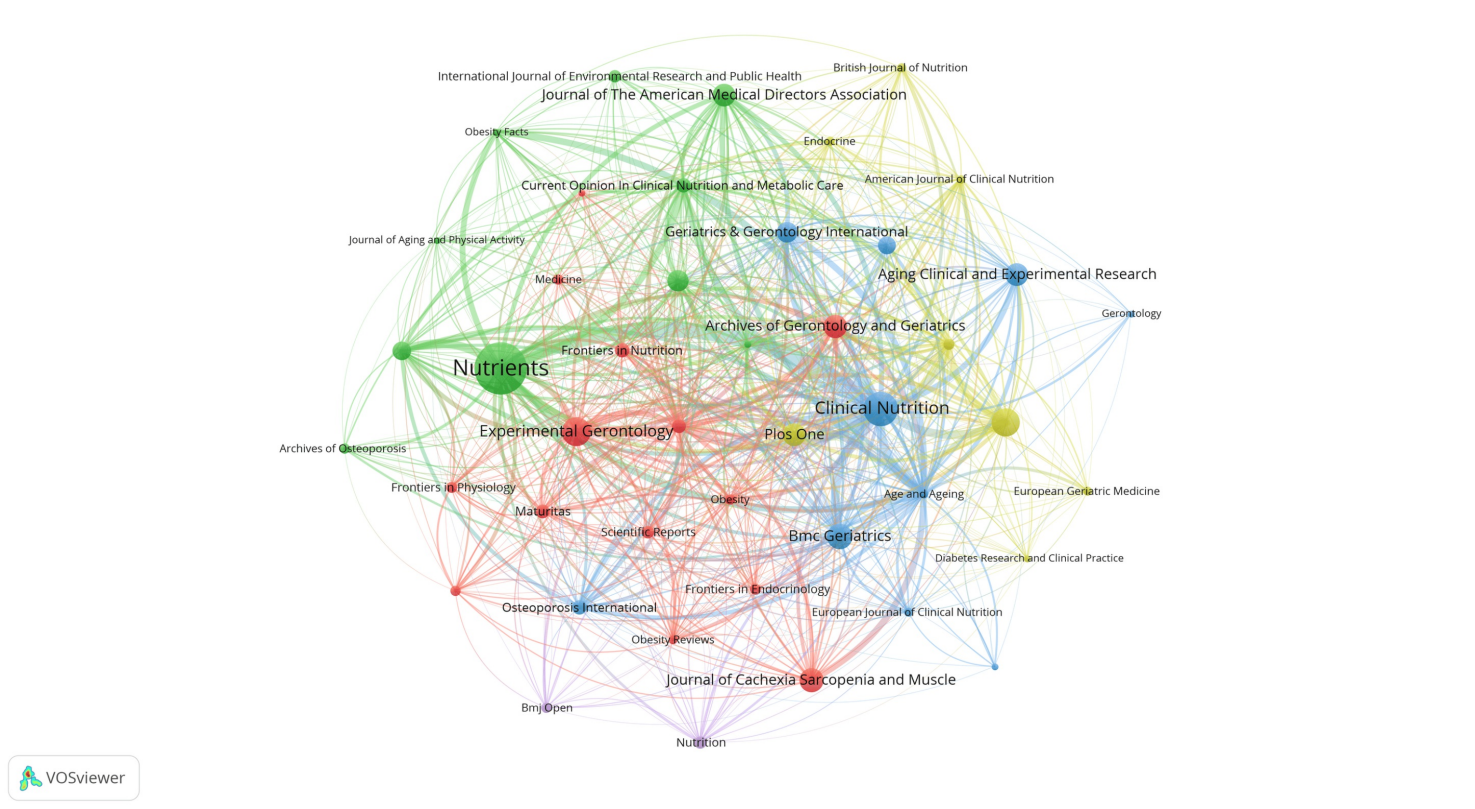
The analysis of journals in the field of sarcopenic obesity in the elderly. (A) The VOSviewer visualization analyzes the collaborative relationships between journals, where nodes represent journals that have published more than 10 documents. The nodes are colored based on their cluster membership, and the size of the node indicates how frequently the journal appears in the network.

**Figure 9:**
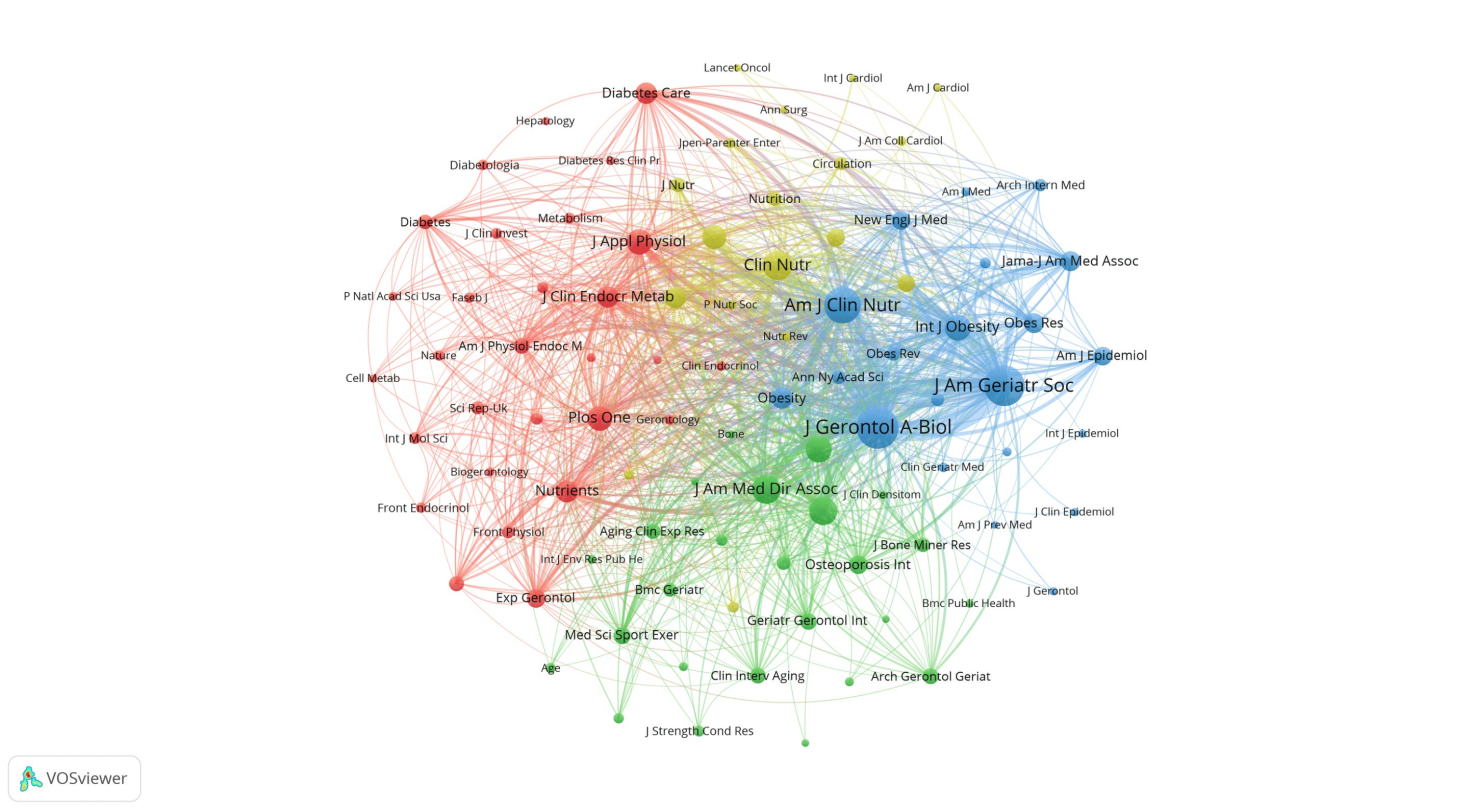
The analysis of journals in the field of sarcopenic obesity in the elderly. The VOSviewer visualization enables the exploration of connections between journals, with node size indicating the frequency of citations and reflecting the significance and influence of the journals in the network.

Figures 8, 9 visually represent the citation and co-citation relationships among the mentioned journals. The journal Nutrients emerges as a central journal in the field, closely linked to publications like Clinical Nutrition and the Journal of the American Medical Directors Association. The research areas covered by these journals are depicted in Figure 9 using distinct colored clusters. For example, journals focusing on nutrition, such as Clinical Nutrition and Nutrition, are grouped in a yellow cluster. Journals centered on physiology and biology, like the Journal of Applied Physiology and the Journal of Clinical Endocrinology and Metabolism, are clustered in red. Publications related to gerontology and obesity, such as Journals of Gerontology Series A: Biological Sciences and Medical Science, Journal of the American Geriatrics Society, and International Journal of Obesity, are highlighted in blue. Journals associated with clinical medicine, such as the Journal of the American Medical Directors Association and Osteoporosis International, form the green cluster.

Keywords analysis

In addition to providing basic information, such as the geographic region, institutions, countries, and authors of an article, keywords represent the main focus or core viewpoints of the research and are a key focus in bibliometric analysis. They precisely reveal the research hotspots and frontiers in a particular field over a specific time period, aiding scholars in predicting future trends. The top 20 keywords based on occurrence frequency and total link strength are presented in Table 5. Keywords with high occurrence frequency include sarcopenia (436 times), obesity (286 times), sarcopenic obesity (230 times), and elderly (215 times). Another important metric is their co-occurrence relationships. This concept not only reflects the overlap in research content but may also unveil potential connections between different research directions, facilitating scholars in exploring new research perspectives.

**Table 5:** Ranking of the top 20 major keywords of sarcopenic obesity in elderly research from 2004 to 2023.

Rank	Keyword	Occurrences	Total link strength	Rank	Keyword	Occurrences	Total link strength
1	Sarcopenia	436	1110	11	Mortality	44	148
2	Obesity	286	840	12	Exercise	43	151
3	Sarcopenic obesity	230	607	13	Insulin resistance	37	107
4	Elderly	215	571	14	Nutrition	31	94
5	Aging	123	366	15	Skeletal muscle	31	84
6	Body composition	115	315	16	Inflammation	30	97
7	Muscle mass	57	172	17	Osteoporosis	30	99
8	Frailty	54	134	18	Malnutrition	29	74
9	Body mass index	53	143	19	Metabolic syndrome	29	81
10	Muscle strength	46	151	20	Skeletal muscle mass	29	66

**Figure 10:**
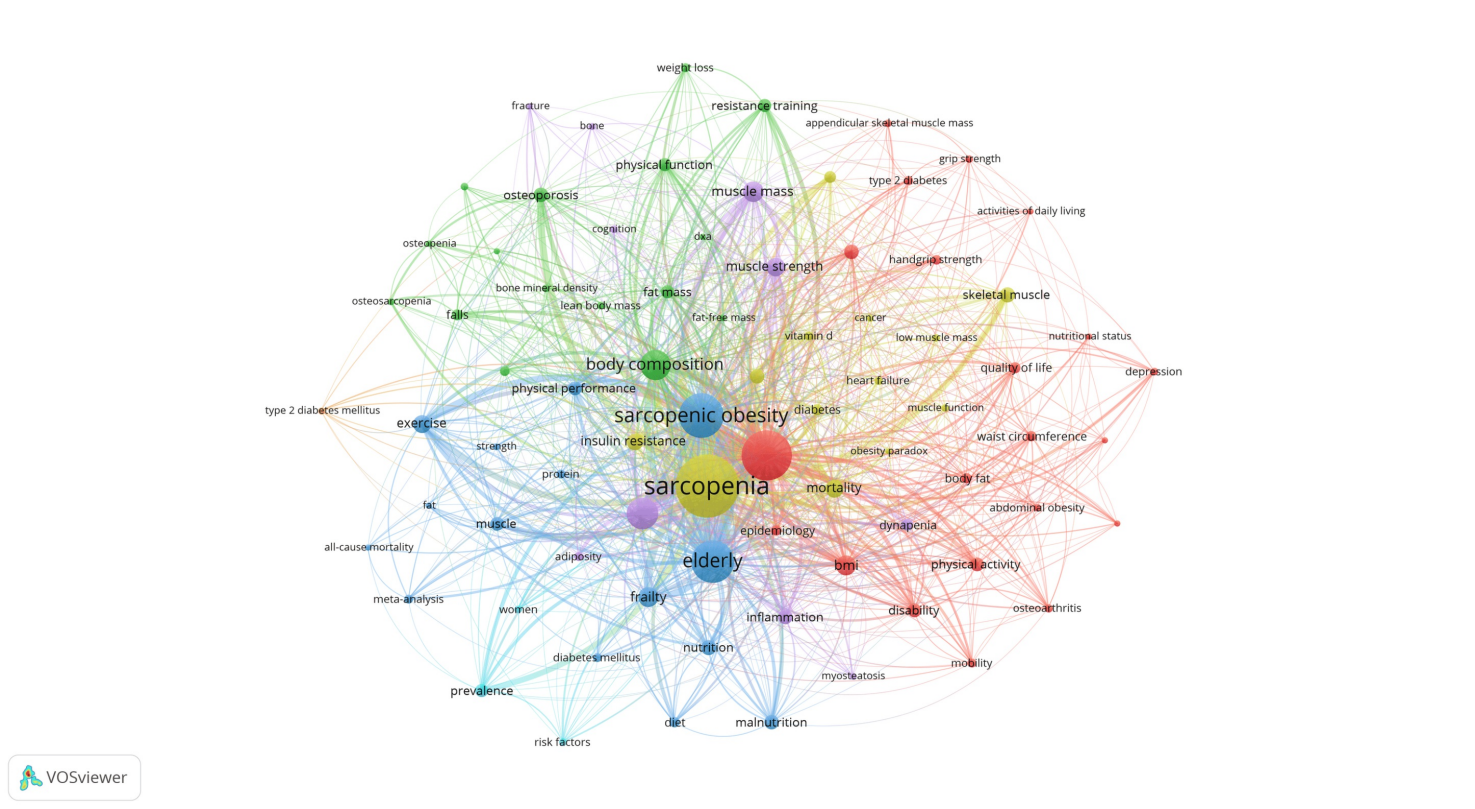
The analysis of keywords in the field of sarcopenic obesity in the elderly. The keyword map of sarcopenic obesity in elderly research visually displays the connections among studied keywords. Nodes, distinguished by various colors, represent different keyword clusters. The size of each node reflects the frequency of co-occurrence, while connections between nodes illustrate relationships among keywords.

**Figure 11:**
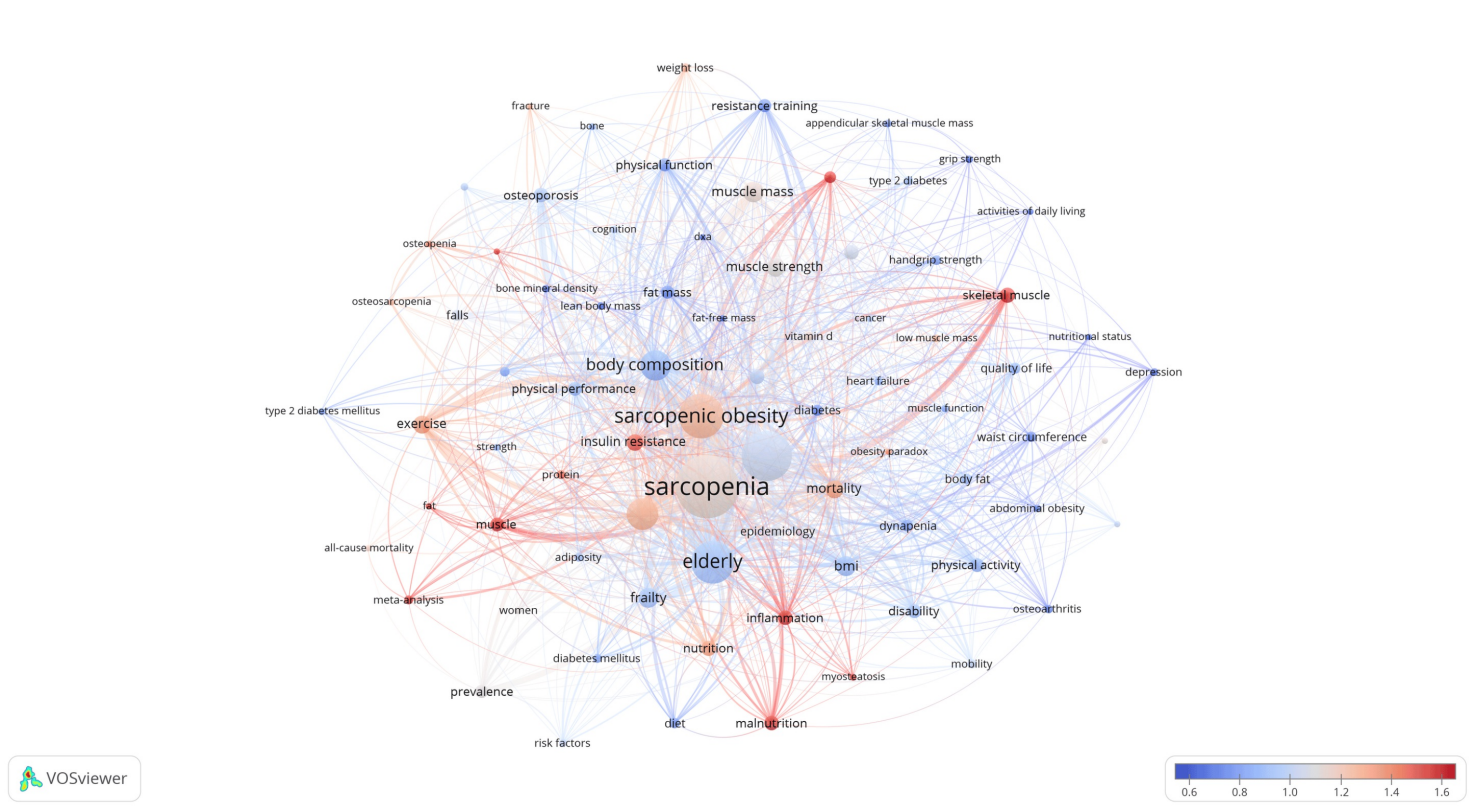
The analysis of keywords in the field of sarcopenic obesity in the elderly. The figure illustrates the recent research contributions of institutions to sarcopenic obesity in the elderly compared to their overall output from 2004 to 2023. A red bias indicates increased influence, while a blue bias suggests decreased activity in the field. The color scale represents the ratio of keywords over the past five years, emphasizing institutions with significant impacts or reduced involvement in this study.

**Figure 12:**
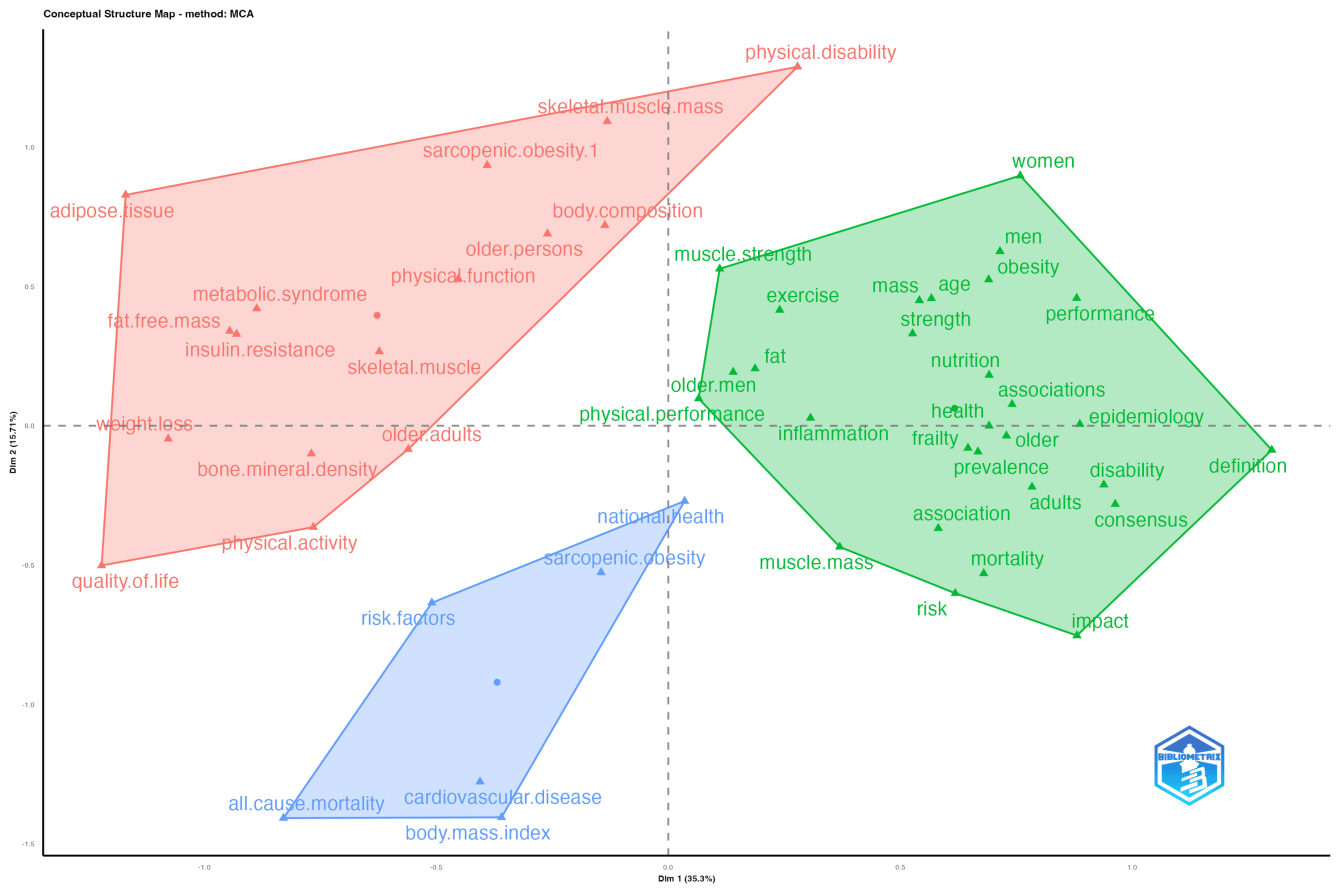
The analysis of keywords in the field of sarcopenic obesity in the elderly. The word map demonstrates the outcomes of automated categorization of important keywords within the field using factorial analysis. It uncovers potential associations among these keywords and provides insights into research areas and application directions they suggest.

Figures 10, 11 depict the co-occurrence relationships and strengths among keywords. The central keyword, sarcopenia, is highlighted in a yellow cluster along with medical terms like diabetes, cancer, and heart failure. The blue cluster includes popular keywords such as sarcopenic obesity and elderly, alongside terms related to physical performance like exercise and muscle. Keywords in the green cluster mainly center around body composition, including fat and bone. In Figure 12, a more intuitive categorization of research areas linked to these keywords is presented. Keywords in the green region describe concepts or factors related to body performance, those in the red region focus on concepts related to body composition and physical function, while the blue region pertains to clinical indicators or diseases related to SO.

Highly cited references analysis

In the domain of research, the number of citations an article receives is a key metric for evaluating its quality and influence. Highly cited articles play a crucial role in advancing research and reflecting current research trends. Table 6 provides an overview of the top 10 most cited articles. The most cited article, "Sarcopenia: European consensus on definition and diagnosis" by Cruz-Jentoft et al. (2010), published in Age and Ageing, is a collaborative effort by the European Working Group on Sarcopenia in Older People (EWGSOP). This article refines the definition of sarcopenia, introduces relevant parameters and diagnostic indicators, and explores its connections with other diseases [12]. The second most cited article, "ESPEN guidelines on definitions and terminology of clinical nutrition" by Cederholm, T et al. (2017) in Clinical Nutrition, systematically categorizes and summarizes concepts and terms related to clinical nutrition, such as sarcopenia, frailty, overweight, and obesity [13]. This standardized classification greatly enhances understanding of clinical nutrition and encourages further exploration within the academic community. Another highly cited article, with over 1000 citations, is "Research agenda for frailty in older adults: Toward a better understanding of physiology and etiology: Summary from the American Geriatrics Society/National Institute on Aging Research Conference on Frailty in Older Adults," authored by Walston, J et al. This article delves into the concept of frailty, exploring the physiological mechanisms of sarcopenia, factors that influence it, its connections to other frailty-related conditions, and its potential as a target for therapeutic interventions [14].

**Table 6:** Ranking of the top 15 major highly cited references of sarcopenic obesity in elderly research from 2004 to 2023.

Rank	Author	Article title	Source title	Cited	Year	Category	DOI
1	Cruz-Jentoft, AJ; Baeyens, JP; Bauer, JM; Boirie, Y; Cederholm, T; Landi, F; Martin, FC; Michel, JP; Rolland, Y; Schneider, SM; Topinková, E; Vandewoude, M; Zamboni, M [12]	Sarcopenia: European consensus on definition and diagnosis	Age and Ageing	7654	2010	Article	10.1093/ageing/afq034
2	Cederholm, T; Barazzoni, R; Austin, P; Ballmer, P; Biolo, G; Bischoff, SC; Compher, C; Correia, I; Higashiguchi, T; Hoist, M; Jensen, GL; Malone, A; Muscaritoli, M; Nyulasi, I; Pirlich, M; Rothenberg, E; Schindler, K; Schneider, SM; de van der Schueren, MAE; Sieber, C; Valentini, L; Yu, JC; Van Gossum, A; Singer, P [13]	ESPEN guidelines on definitions and terminology of clinical nutrition	Clinical Nutrition	1127	2017	Article	10.1016/j.clnu.2016.09.004
3	Walston, J; Hadley, EC; Ferrucci, L; Guralnik, JM; Newman, AB; Studenski, SA; Ershler, WB; Harris, T; Fried, LP [14]	Research agenda for frailty in older adults: toward a better understanding of physiology and etiology: summary from the American Geriatrics Society/National Institute on Aging Research Conference on Frailty in Older Adults	Journal of the American Geriatrics Society	1066	2006	Article	10.1111/j.1532-5415.2006.00745.x
4	Deutz, NEP; Bauer, JM; Barazzoni, R; Biolo, G; Boirie, Y; Bosy-Westphal, A; Cederholm, T; Cruz-Jentoft, A; Krznariç, Z; Nair, KS; Singer, P; Teta, D; Tipton, K; Calder, PC [15]	Protein intake and exercise for optimal muscle function with aging: Recommendations from the ESPEN Expert Group	Clinical Nutrition	869	2014	Article	10.1016/j.clnu.2014.04.007
5	Stenholm, S; Harris, TB; Rantanen, T; Visser, M; Kritchevsky, SB; Ferrucci, L [16]	Sarcopenic obesity: definition, cause and consequences	Current Opinion in Clinical Nutrition and Metabolic Care	735	2008	Review	10.1097/MCO.0b013e328312c37d
6	Kalyani, RR; Corriere, M; Ferrucci, L [17]	Age-related and disease-related muscle loss: the effect of diabetes, obesity, and other diseases	Lancet Diabetes & Endocrinology	617	2014	Review	10.1016/S2213-8587(14)70034-8
7	Baumgartner, RN; Wayne, SJ; Waters, DL; Janssen, I; Gallagher, D; Morley, JE [18]	Sarcopenic obesity predicts instrumental activities of daily living disability in the elderly	Obesity Research	614	2004	Article	10.1038/oby.2004.250
8	Zamboni, M; Mazzali, G; Fantin, F; Rossi, A; Di Francesco, V [19]	Sarcopenic obesity: A new category of obesity in the elderly	Nutrition, Metabolism and Cardiovascular Diseases	561	2008	Review	10.1016/j.numecd.2007.10.002
9	Batsis, JA; Villareal, DT [20]	Sarcopenic obesity in older adults: aetiology, epidemiology and treatment strategies	Nature Reviews Endocrinology	555	2018	Review	10.1038/s41574-018-0062-9
10	Delmonico, MJ; Harris, TB; Lee, JS; Visser, M; Nevitt, M; Kritchevsky, SB; Tylavsky, FA; Newman, AB [21]	Alternative definitions of sarcopenia, lower extremity performance, and functional impairment with aging in older men and women	Journal of the American Geriatrics Society	543	2007	Article	10.1111/j.1532-5415.2007.01140.x
11	Narici, MV; Maffulli, N [22]	Sarcopenia: characteristics, mechanisms and functional significance	British Medical Bulletin	458	2010	Article	10.1093/bmb/ldq008
12	Zamboni, M; Mazzali, G; Zoico, E; Harris, TB; Meigs, JB; Di Francesco, V; Fantin, F; Bissoli, L; Bosello, O [23]	Health consequences of obesity in the elderly: a review of four unresolved questions	International Journal of Obesity	442	2005	Review	10.1038/sj.ijo.0803005
13	Srikanthan, P; Hevener, AL; Karlamangla, AS [24]	Sarcopenia exacerbates obesity-associated insulin resistance and dysglycemia: findings from the National Health and Nutrition Examination Survey III	PLoS One	400	2010	Article	10.1371/journal.pone.0010805
14	Lim, S; Kim, JH; Yoon, JW; Kang, SM; Choi, SH; Park, YJ; Kim, KW; Lim, JY; Park, KS; Jang, HC [25]	Sarcopenic obesity: Prevalence and association with metabolic syndrome in the Korean Longitudinal Study on Health and Aging (KLoSHA)	Diabetes Care	398	2010	Article	10.2337/dc10-0107
15	Kim, TN; Park, MS; Yang, SJ; Yoo, HJ; Kang, HJ; Song, W; Seo, JA; Kim, SG; Kim, NH; Baik, SH; Choi, DS; Choi, KM [26]	Prevalence and determinant factors of sarcopenia in patients with type 2 diabetes	Diabetes Care	387	2010	Article	10.2337/dc09-2310

**Figure 13:**
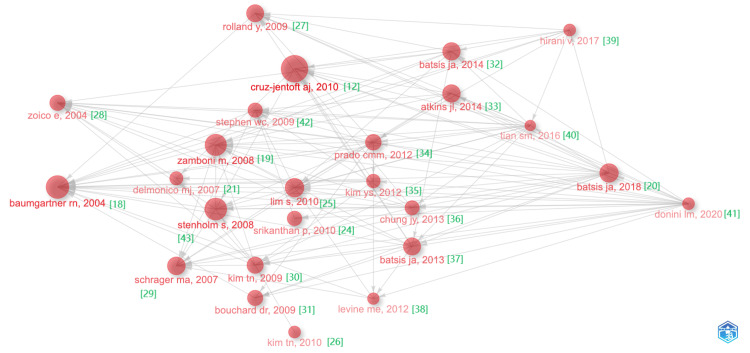
The analysis of highly cited references in the field of sarcopenic obesity in the elderly. A visual representation illustrates the connection among the top 25 extensively referenced sources. The size of each node corresponds to the level of citation intensity, and the nodes are arranged from left to right in chronological order of citation occurrences, with the most recently cited sources placed farthest to the right.

**Figure 14:**
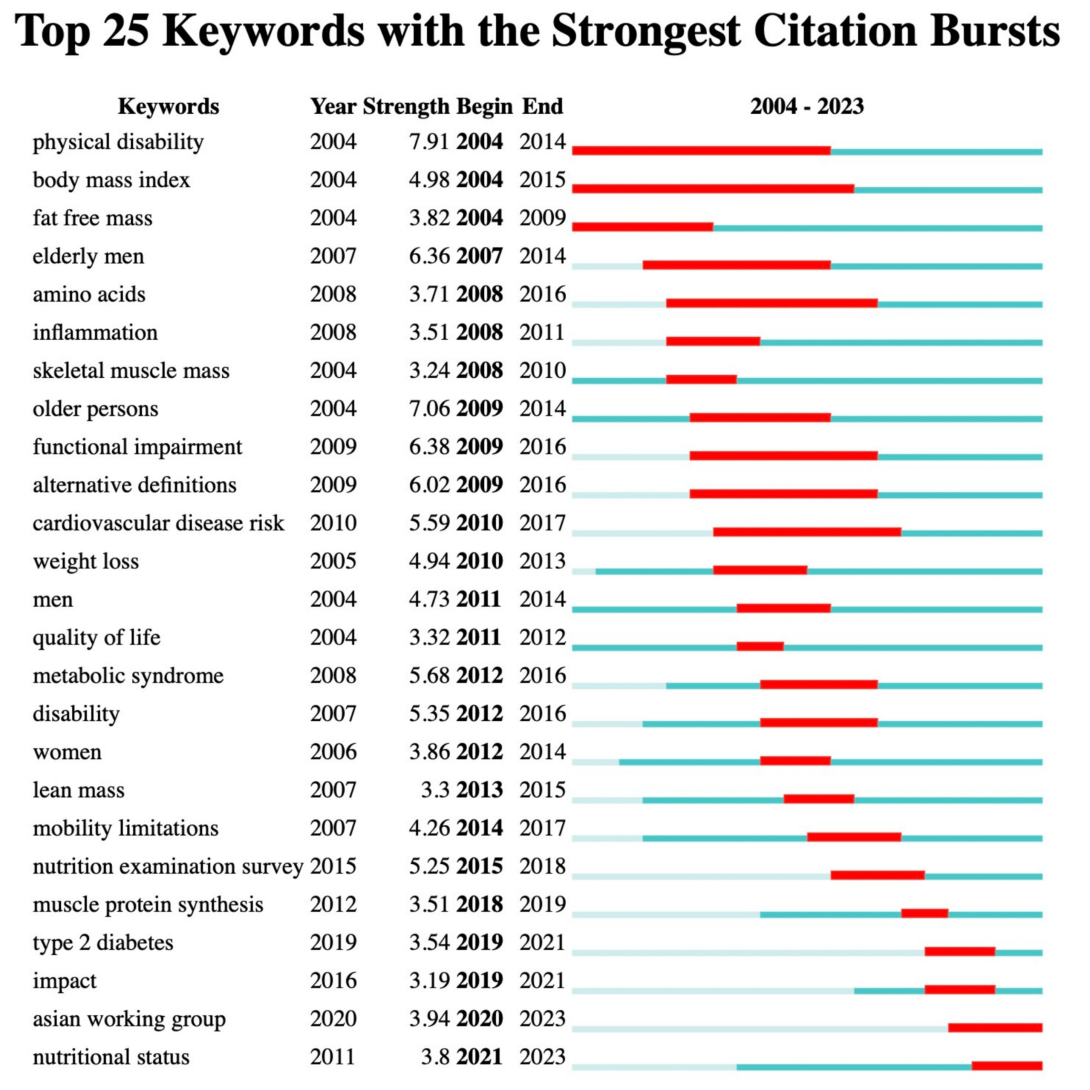
The analysis of highly cited references in the field of sarcopenic obesity in the elderly. The diagram depicts 25 primary references with noticeable bursts of citations, represented by red spikes on the timeline. These spikes indicate rapid increases in citation counts, highlighting significant moments of important questions or solutions emerging in the field.

The relationships and trends among highly cited articles are demonstrated in Figures 13, 14. The connecting lines in Figure 13 show the connections between these influential publications [12,18-21,24-42]. Notably, the article "Sarcopenic obesity predicts instrumental activities of daily living disability in the elderly" by Baumgartner, RN et al. in 2004 [18] has been a key work in the field of SO in the elderly over the last two decades. This publication has influenced subsequent research and inspired the comprehensive article "Sarcopenic obesity in older adults: aetiology, epidemiology and treatment strategies" by Batsis, JA et al. [20]. In addition to the developmental context, the significant increase in citation counts is also a noteworthy aspect. As shown in Figure 14, the articles with the highest burst intensities in the timeline are the two works by Cruz-Jentoft et al., published in 2010 and 2019, titled "Sarcopenia: European consensus on definition and diagnosis" (41.38) [12] and "Sarcopenia: revised European consensus on definition and diagnosis" (39.63) [43]. On the other hand, the six articles at the bottom of the timeline are still showing bursts, indicating that the topics addressed in these articles are currently popular in research and offer scholars insights into the latest trends and potential research directions.

## Discussion

SO is characterized by a concurrent decline in muscle mass and function, coupled with a rise in adipose tissue. This issue is becoming more concerning among elderly individuals because of its significant health consequences, including effects on longevity, associated conditions, and vulnerability to age-related disorders. The complex pathophysiology of SO entails a multifaceted interplay among muscle, fat tissue, hormonal fluctuations, inflammation, oxidative stress, and lifestyle factors [44]. Our study involved the analysis of 985 articles on SO in the Web of Science Core Collection. The literature was analyzed in depth by country/region, institution, journal, author, and keywords. Bibliometrix R software and CiteSpace were utilized to explore the knowledge structure, research hotspots, and emerging trends in this field. The findings aim to provide a foundation for the development of preventive and therapeutic strategies for this complex condition.

The interest in SO among older adults is growing, with the number of citations in this field increasing each year, reaching 127 in 2022. Among the 79 countries and regions that have conducted research on SO in older individuals, the United States stands out for its high publication output and substantial international collaborations. There has also been a noteworthy rise in the number of publications from South Korea and China, likely influenced by the increasing elderly population in these nations. Three of the top 10 institutions with the most publications on this topic are from both the United States and South Korea. Italy emerges as a key focus of international research, ranking second only to the US in total citation count and link strength. Both countries have shown strong publication outputs and link strength in this area. Additionally, all of the leading 10 journals in terms of publication and citation counts related to SO in older adults fall under the Q2 category or higher, indicating significant interest from both the public and scientific community in this field.

Keywords with close co-occurrence relationships can be categorized into three main clusters. The first cluster delves into the endocrinological and metabolic aspects of SO in older adults, encompassing metabolic syndrome, adipose tissue, fat-free mass, body composition, and insulin resistance. The second cluster revolves around the conceptualization of SO in older adults, covering aspects like definition, consensus, pathophysiology (inflammation), and the clinical significance of SO in older adults, such as its impact on frailty, disability, and mortality. The third cluster primarily addresses risk factors and clinical implications of SO in older adults, specifically focusing on all-cause mortality and cardiovascular disease.

The pathogenesis of SO is multifaceted, involving aging, a sedentary lifestyle, poor dietary choices, insulin resistance, inflammation, and oxidative stress. These factors collectively contribute to the decline in muscle mass and function, alongside an increase in fat mass. Among the top 20 keywords associated with the pathogenesis of SO are aging, insulin resistance, inflammation, and nutrition/malnutrition. The relationship between inflammation and SO has been a prominent subject of research in recent years. Muscle and adipose tissue share common inflammatory pathways, through which peptides are produced in an autocrine and paracrine manner to facilitate intercellular signaling. These pathways are interconnected, contributing to the pathogenesis of SO [45]. The secretion of peptides by adipocytes is closely tied to the size of these cells, with larger adipocytes producing higher levels of pro-inflammatory adipokines and lower levels of anti-inflammatory adipokines. Weight gain can lead to adipocyte enlargement, disrupting the balance between pro- and anti-inflammatory adipokines. Additionally, as individuals age, adipocytes tend to exhibit more pro-inflammatory characteristics. Obesity triggers the activation of macrophages, mast cells, and T lymphocytes, resulting in the production of various adipokines and cytokines like tumor necrosis factor-α (TNF-α), interleukins, and interferon-γ. This immune response causes infiltration of immune cells and sets off an inflammatory cascade. These inflammatory factors are not limited to adipose tissue, potentially exacerbating inflammation and oxidative stress. Impaired mitochondrial beta-oxidation can increase lipid peroxidation, leading to the accumulation of lipid intermediates and reactive oxygen metabolites. This, in turn, heightens insulin resistance, inflammation, oxidative stress, and myocytic lipotoxicity, ultimately contributing to myocyte dysfunction and apoptosis [46]. Muscle biopsies from obese individuals have revealed upregulated expression of chemokine genes that hinder myocyte differentiation and proliferation, providing further evidence for this perspective [47].

Our study identified that the hotspots regarding SO interventions were "nutrition" and "exercise." The key objectives in treating SO are to combine exercise and nutritional interventions with the aim of creating a negative energy balance to reduce adipose tissue (improving adipose markers and reducing inflammatory parameters) while preserving and ideally increasing muscle mass and function [48]. Energy restriction activates cellular stress response elements, improves autophagy, and alters apoptosis and hormone balance. However, caution should be exercised when implementing low-calorie diets for weight loss to prevent compromising muscle mass and strength. Some scholars suggest that an energy restriction of approximately 500 kcal/d (1 kcal = 4.18 kJ) can effectively promote weight loss, is safe, and can counteract the reduction in non-fat mass. A reasonable weight loss target should not exceed 5% to 8% of the initial weight. Moderate weight loss (around 5%) has been shown to reduce muscle fat infiltration and enhance muscle function. Therefore, patients following a low-calorie diet may have increased protein requirements, and protein intake should be carefully managed to preserve fat-free mass and enhance muscle strength. The optimal type and quantity of protein supplements have yet to be determined, but it is recommended to supplement essential amino acids like leucine, arginine, and cysteine. As for the amount of protein supplements, it is advised to consume 1.0~1.2 g·kg-1·d-1, with some scholars suggesting 1.2~1.6 g·kg-1·d-1. In individuals with renal impairment, the dosage should be adjusted accordingly. Evidence suggests that specific nutrients such as beta-hydroxymethylbutyrate, vitamin D, creatine, omega-3 fatty acids, and low calcium levels can enhance protein anabolism, improve muscle mass, increase sensitivity to anabolic signals, and reduce fat accumulation while promoting lipid oxidation. Further research is necessary to elucidate the precise benefits of these nutrients, as well as to identify their optimal combinations and dosages [49].

Exercise has a multitude of beneficial effects on individuals with SO. It can influence hormone balance, reduce oxidative stress, lower inflammatory responses, promote mitochondrial synthesis, alter immune and motor functions, increase muscle oxidative capacity, enhance muscle protein synthesis, and enhance insulin sensitivity. Simply losing weight without including exercise may result in a reduction of fat-free mass, worsening the condition. However, incorporating exercise along with energy restriction can aid in preventing the loss of fat-free mass [50].

The optimal form of exercise for SO patients is yet to be definitively determined. However, current data indicate that a combination of resistance exercise and aerobic exercise may be the most effective. Resistance training has been shown to be more beneficial in combating sarcopenia, while aerobic training is more effective in addressing obesity. A six-month randomized controlled trial involving obese older adults found that the combined exercise group showed greater improvements in physical performance tests and quality of life compared to groups that only engaged in resistance or aerobic exercises. This group also experienced greater strength gains, increased lean mass, and reduced resistance. Although limited, data on alternative forms of exercise such as whole-body electrical stimulation, vibration, yoga, and tai chi suggest that they may also be effective for SO patients, particularly for those with physical disabilities or mobility issues [51,52].

There is currently a lack of studies on interventions for SO in older adults among the top 15 most highly cited references. This is partly due to the fact that the majority of these highly cited references were published before 2018. While it is well established that resistance exercise can improve skeletal muscle function in SO, the American College of Sports Medicine recommends a strength training program to be conducted at least twice a week on non-consecutive days, with one set of eight to 12 repetitions for healthy adults and 10-15 repetitions for older and frail individuals [53]. However, there remains a scarcity of clinical studies focused on a comprehensive intervention tailored specifically to SO in older adults. A meta-analysis of 15 studies on the effects of exercise and nutritional intervention on body composition, metabolic health, and physical performance in adults with SO found that aerobic exercise reduced body weight and fat mass (FM). Resistance exercise (RE) decreased FM and improved grip strength, while the combination of aerobic exercise and RE decreased FM and improved walking speed. Nutritional interventions, particularly low-calorie high-protein (LCHP) diets, reduced FM but did not impact muscle mass and grip strength. Despite exercise training being crucial for improving body composition and physical performance in individuals with SO, nutritional intervention with LCHP only decreased FM without enhancing physical performance [54]. Elderly individuals with SO commonly exhibit a variety of complex conditions and increased heterogeneity, including multiple chronic illnesses and geriatric syndromes. Performing interventional research on this diverse group with SO presents many challenges.

While previous bibliometric analyses have examined SO, there remains a notable gap in research on SO among the elderly population. This study stands out as the first bibliometric analysis focusing on research related to SO specifically in older adults. The findings from this analysis are essential for gaining insights into current research trends, key areas of interest, and potential future research directions concerning SO in the elderly. Our bibliometric analysis underscores that SO among the elderly is a significant research area that encompasses geriatrics, gerontology, endocrine and metabolic diseases, and clinical nutrition. While academic research has shed light on SO, numerous clinical studies have underscored the efficacy of exercise and nutritional interventions in addressing obesity, sarcopenia, and the combination of both in adults. Nevertheless, there remains a dearth of clinical research and evidence-based medicine on effectively managing obese sarcopenia in the elderly. Given the vast heterogeneity and complexity of the elderly population, significant challenges persist in tackling this issue. In clinical settings, it is imperative for healthcare providers to acknowledge the presence and detrimental effects of obese sarcopenia and not overlook it, as doing so could lead to overlooking a critical issue. For elderly individuals dealing with both obesity and sarcopenia, it is recommended to conduct a comprehensive geriatric assessment (CGA). By utilizing the assessment results along with the patient's comorbidities, physical functioning, social support, and life expectancy, a geriatric interdisciplinary team (GIT) can develop personalized interventions to improve the patient's quality of life and overall prognosis.

This investigation has several limitations. While the study heavily relies on the Web of Science Core Collection database for its vast literary resources and reliable information, there is a risk of a potentially incomplete literature search. To improve coverage, it would be beneficial to include additional databases such as PubMed and Scopus. Furthermore, the search strategy limited to the terms "sarcopenic obesity" may have excluded relevant publications, introducing a risk of selection bias. The study exclusively focuses on English-language literature, potentially overlooking valuable non-English-language literature. Additionally, the bibliometric analysis does not assess the quality of the searched literature. Lastly, the study only considers literature published between 2004 and 2023, suggesting that researchers interested in the field should analyze newly published research since 2024.

## Conclusions

The analysis indicates a significant increase in global research focusing on SO in older adults, a key area of study spanning geriatrics, gerontology, endocrine and metabolic diseases, and clinical nutrition. The United States leads in this research domain. While academic investigations have illuminated aspects of SO, there is a noticeable lack of clinical research and evidence-based medicine on effectively managing obese sarcopenia in the elderly. With the aging population, there is an urgent need for coordinated efforts to reduce adverse health outcomes associated with geriatric SO. Tailored interventions for older adults with SO should be a priority. Our study can serve as a guide for SO research in older adults, aiding researchers in identifying crucial information and senior experts in expanding collaboration networks or designing future clinical trials.
