# Correction: Mapping Knowledge Landscapes and Emerging Trends of Sarcopenic Obesity in Older Adults: A Bibliometric Analysis From 2004 to 2023

**DOI:** 10.7759/cureus.c190

**Published:** 2024-08-20

**Authors:** Ning Zhang, Xuan Qu, Haokang Zhou, Lin Kang

**Affiliations:** 1 Department of Geriatrics, Peking Union Medical College Hospital, Peking Union Medical College, Chinese Academy of Medical Sciences, Beijing, CHN; 2 Department of Internal Medicine, Peking Union Medical College Hospital, Peking Union Medical College, Chinese Academy of Medical Sciences, Beijing, CHN

This article has been corrected to reflect that Dr. John Batsis is affiliated with University of North Carolina at Chapel Hill and not Duke University as originally stated. The authors and journal deeply regret that this error was not identified and addressed prior to publication.

